# Reciprocity heightens academic performance in elementary school students

**DOI:** 10.1016/j.heliyon.2022.e11916

**Published:** 2022-12-05

**Authors:** Cristian Candia, Melanie Oyarzún, Victor Landaeta, T. Yaikin, Cecilia Monge, César Hidalgo, Carlos Rodriguez-Sickert

**Affiliations:** a Data Science Institute, Facultad de Ingeniería, Universidad del Desarrollo, Las Condes, 7610658, Chile; b Northwestern Institute on Complex Systems (NICO), Northwestern University, Evanston, IL 60208, USA; c Centro de Investigación en Complejidad Social (CICS), Facultad de Gobierno, Universidad del Desarrollo, Chile; d Feedback Communications, Vitacura, Chile; e ANITI Chair, University of Toulouse, Toulouse, France; f Alliance Manchester Business School, University of Manchester, Manchester, UK; g School of Engineering and Applied Sciences, Harvard University, USA

**Keywords:** Academic performance, Peer interaction, Primary education, Reciprocity, Nonanonymous social dilemma, Experimental game theory

## Abstract

Social relationships are pivotal for human beings. Yet, we still lack a complete understanding of the types and conditions of social relationships that facilitate learning among children. Here, we present the results of a study involving 855 elementary school children from 14 different public schools in Chile designed to understand their social learning strategies in classrooms. We mapped students' social relationships using a behavioral experiment–a non-anonymous social dilemma–that allows us to measure cooperation and infer reciprocal and asymmetrical relationships between peers. We implemented the experiment synchronously in each classroom using networked tablets and a friendly user interface to mitigate cognitive barriers and boost students' engagement. Using regression models, we found a positive and significant association between reciprocity and academic performance. This result holds after controlling for class attendance, sex, parents’ education, social status, individual cooperative dispositions, and fixed effects per class group. Finally, using a difference-in-difference framework, we found robust evidence that reciprocity heightens academic performance by comparing two consecutive academic semesters. This effect is heterogeneous and is considerably more prominent for the top 20% students experiencing higher levels of reciprocity in their social relationships. We expect these results to inform cooperative learning interventions in elementary education.

## 1 Introduction

“No significant learning can occur without a significant relationship,” in this statement, Dr. James Comer from Yale University clearly expresses the critical role of social relationships between students and their peers, teachers, friends, and family in learning. Certainly, this sentence does not mean that we cannot learn from people with no direct relationships, yet, knowledge and experiences acquired from meaningful relationships are remembered and applied more than others.

In recent decades, several studies emphasize a significant association between students' social relationships and their academic performance at different ages (Baldwin et al., 1997; Caprara et al., 2000; Bruun and Brewe, 2013; Gašević et al., 2013; Blansky et al., 2013; Ivaniushina and Alexandrov, 2018; Stadtfeld et al., 2019; Kassarnig et al., 2018; Berthelon et al., 2019; Pulgar et al., 2020; Candia et al., 2022a, Candia et al., 2022b; Pulgar et al., 2022a, Pulgar et al., 2022b; Candia et al., 2019; Smirnov and Thurner, 2017). For instance, academic performance correlates positively with social capital–the individuals’ network of connections and tacit cooperation (Halpern, 2005)–among college students in online degree programs (Gašević et al., 2013), and with the flow of online and offline communication among undergraduates (Kassarnig et al., 2018).

Literature has shown positive externalities–peer effects–that are known to be pivotal for social learning (Henrich, 2015; Gil-White and Henrich, 2001; Pentland, 2015; Johnson and Johnson, 1987; Roger and Johnson, 1994) and play a key role in academic outcomes (Kassarnig et al., 2018; Blansky et al., 2013; Sacerdote, 2011; Biancani and McFarland, 2013; Davies, 2018). As a matter of fact, the teaching strategy has a significant impact on how well students capture the results of their social interactions within a certain social setting.

Indeed, how students capture the effects of their social relationships within a particular social environment largely depends on the teaching strategy (Pulgar et al., 2020) and the social structure of their cooperative relationships (Calvó-Armengol et al., 2009). For instance, social learning can be understood as a natural form of pedagogy, where cognitive mechanisms enable the transmission of cultural knowledge through imitation and communication (Csibra and Gergely, 2011; Pulgar et al., 2022a, Pulgar et al., 2022b). What distinguishes this natural pedagogy from other types of social learning, e.g., prestige-biased social learning (Henrich and Gil-White, 2001; McFarland et al., 2014), is that requires both the disposition for learning from the “student-role subject,” but the readiness of sharing their knowledge from the "teacher-role subject" (Csibra and Gergely, 2011). Therefore, from a game-theoretical point of view, a pedagogic act between peers (Rohrbeck et al., 2003) qualifies as an act of cooperation in a traditional social dilemma, a scenario in which individual and collective interests collide because there are incentives to maximize individuals’ payoffs that generates a sub-optimal collective performance (Kollock, 1998).

Of our particular interest, studying the social factors that contribute to academic performance in elementary school students is relevant because long-term returns to education depend mainly on early learning outcomes–path dependency (Claessens and Engel, 2013; Dickens et al., 2006; Restuccia and Urrutia, 2004). However, the effect of social relationships on academic performance has been understudied among elementary school children, mainly because of the methodological difficulties in extracting experimental social information for such a young population.

Mapping the underlaying social networks driven social relationships and cooperative dynamics requires multidimensional instruments. The first documented mapping of social relationships are Moreno's sociograms (Moreno, 1934), obtained surveying students about who they like or dislike to spend time with and who their friends are (Coie et al., 1990; Mouw, 2006; Neal, 2007; McCormick and Cappella, 2015; Ivaniushina and Alexandrov, 2018). Yet, survey-based social network mapping may exacerbate different types of biases in primary school students (Leeuw, 2011), such as the social desirability bias (Van de Mortel et al., 2008) (over-reporting of socially desirable behavior); cognitive barriers (difficult to establish that subjects fully understand the questions) (Borgers et al., 2000); and lack of engagement (length or unfriendliness of instruments generate poor answers) (Banister and Booth, 2005; Barker and Weller, 2003; Fleer and Ridgway, 2014; Kyritsi, 2019) associated with the implementation of self-report based instruments.

To tackle these biases, we implemented a game-theory-based experiment in which all the students of a given class play a dyadic social dilemma with each classmate. Social dilemmas have been studied from a theoretical (Boyd et al., 2003; Nowak, 2006; Guzmán et al., 2007; Delton et al., 2011; Santos et al., 2012; Capraro and Perc, 2021; Perc, 2016) and an experimental point of view (Rivera-Hechem et al., 2021; Fehr and Gächter, 2000; Fehr and Leibbrandt, 2011), which have contributed enormously to the understanding of the dynamics of human cooperation. A new body of literature has shown that cooperative social norms that are prevalent in the real world can penetrate laboratory behavior (Camerer, 2011). Individuals who are more cooperative in the real world also behave more cooperatively in the lab (Carpenter and Seki, 2011; Algan et al., 2013); and groups who achieve higher levels of cooperation in the real world also achieve higher levels of aggregate cooperation when playing a social dilemma in the lab (Fehr and Leibbrandt, 2011; Gelcich et al., 2013; Hopfensitz and Miquel-Florensa, 2017). The social domain of all of these experimental studies ranges from the fishers of Toyama Bay (Fehr and Leibbrandt, 2011) through the exploitation of benthic resources on the Chilean coast (Rivera-Hechem et al., 2021) to the Wikipedians (Algan et al., 2013). While all the above-referenced studies involved anonymous interactions, recent studies have shown that non-anonymous interaction increases cooperation in contrast with anonymous interaction, suggesting that pre-existing social connections affect laboratory behavior (Wang et al., 2017; Conte, 2022).

In this paper, we implemented a game played on a networked tablet set using a friendly drag-and-drop interface. This methodological approach facilitates the behavioral mapping of social interactions to uncover cooperative relationships by settling elementary school students in a familiar and ecological interactive environment. The advantage of using game theory to map the social network is twofold: first, due to the non-anonymous character of the game, it allows us to capture in a more comprehensive way the nature of cooperative relationships (Wang et al., 2017; Conte, 2022) among students who, in most cases, have been together in the same class group for more than three years; and second, the interactive nature of the game in which different actions lead to different payoffs mitigates the biases related to survey-based instruments (Camerer and Hogarth, 1999). In short, we use a behavioral measure based on a non-anonymous dyadic social dilemma to map social relationships among elementary school students. Specifically, we focus on the effect of students' reciprocity in each classroom's cooperative network–mapped from the non-anonymous video game played by 946 children aged 9 to 11 from 45 different classrooms in 14 Chilean public schools–on their academic performance.

Here, we will study the following question: Do students who participate in more mutually cooperative relationships increase their GPAs more than other students? We will provide quantitative evidence for the hypothesis that reciprocity among elementary school students positively affects academic performance. A detailed description of the game-theoretic experiment and its interface, data and methods is provided in the next section, followed by an analysis of the results and the identification strategy for establishing outcomes. Then, a discussion of the implications, limitations and suggestions for future research is provided.

## 2 Methods

### 2.1 Sample

We collected experimental data from 855 students (between the 3rd and 5th grade) with an average age of 10.16±1.18 years old (57.5% were females). Data collection involved 14 different public schools and a total of 45 classrooms (see Supplementary Methods 1 for a descriptive table for each classroom). We note that students have a common history together because they have been member of the same class group for more than three years. Our study period spans from July to December 2017. The experiment was run at the beginning of the first semester (July–August 2017). We measured academic performance and attendance in July 2017 and in December 2017. Form the administrative records, we also collected gender and educational level of the student's parent or guardian as control variables. Finally, we note that 86 students do not have the data for the variable “Tutor complete secondary school.” Hence, we studied the 769 students with complete information.

Our data collection methods and the experimental protocol were approved on May 5th, 2016, by the Institutional Research Ethics Committee of Universidad del Desarrollo, and informed consent was obtained from all participants’ tutors from this experiment.

### 2.2 The game

**Figure 1:**
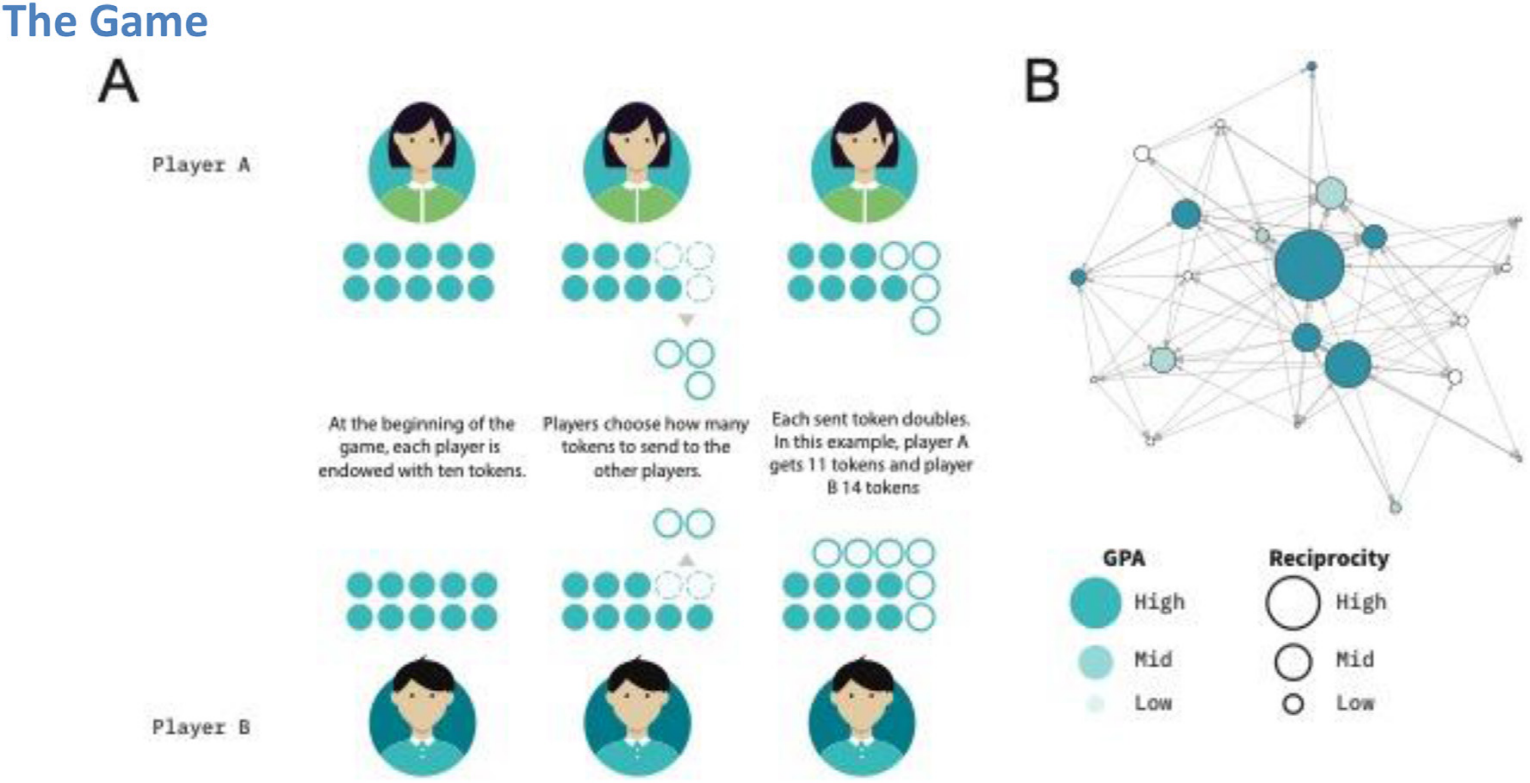
(A) Experimental game. Students play a social dilemma; an example of a dyadic interaction: (i) both players are endowed with ten tokens. (ii) Simultaneously, player A sends three tokens, and player B sends two tokens. (iii) After sent tokens are doubled, Player A receives four tokens, and player B receives six tokens. (B) Visual correlation pattern between GPA and Reciprocity. The figure shows a student interaction network for a single class group using observed behaviors in the game. Each node represents one student, and the directed edges connecting them represent fully cooperative interactions in which at least one individual sent ten tokens. The node size is proportional to the average reciprocity (network reciprocated weight, see Table 1), and colors represent GPA. We observe that the darker the color, the greater the node size suggesting a positive correlation between GPA and reciprocity.

To measure relational cooperation, we implement a modified Prisoner's Dilemma using a friendly user interface on tablet computers (Figure 1A). Our design involves two modifications concerning the standard experimental design. First, the interaction is non-anonymous. In each round, students know who their counterparts are. Under standard game-theoretic experimental protocols involving anonymous interaction, networks elicited in the lab emerge from scratch, mainly through assortative interaction between anonymous players (Goeree et al., 2009). However, we departed from the standard protocol to capture the nature of pre-existing relationships and considered non-anonymous interactions. As a result, students' cooperative decisions are influenced by a variety of factors, including their prosocial tendencies (or lack thereof) as well as their shared experiences and history (Wang et al., 2017).

Second, rather than deciding whether to cooperate, students can implement different levels of cooperation by sending a positive amount of tokens or choosing not to cooperate and keeping all of their tokens. In each class group, students are paired at random and start each round with ten tokens. Then, students decide secretly and simultaneously the number of tokens to keep for themselves and to send to their peers. After each sent token is doubled, students end the round with the number of tokens they kept plus the number of received tokens. The game ends once every possible pair of students played one and only one round of the game. Thus, we created a social dilemma where individuals’ incentives (keep all tokens) face social incentives (give tokens to their peers who share a history and common past).

Figure 1B depicts an example of a mapped cooperative network of a single class group. Nodes represent students and links represent fully cooperative relationships (students that sent ten tokens to a peer). We can visually observe a correlation pattern between GPA (color) and reciprocity (node size).

### 2.3 Network measures

We quantified individuals’ cooperation and reciprocity in their classrooms using network measures. We define a weighted adjacency matrix for each classroom $w_{ij}$, representing the number of sent tokens from student i to student j. Table 1 shows the network metrics used in this study.

**Table 1:** Network measures. $w_{ij}$ corresponds to the number of tokens sent from i to j. In PageRank centrality, d represents a dumping factor (d=0.85 (Page et al., 1999)), and N is the number of students in each classroom.

Network measure	Social Capital	Formula
Average in-degree	Average received cooperation	$r_{i}=\frac{1}{N}\sum_{j\neq i}w_{ji}$
Average out-degree	Average sent cooperation	$s_{i}=\frac{1}{N}\sum_{j\neq i}w_{ij}$
Reciprocated weight	Reciprocated cooperation	$R_{i}=\frac{1}{N}\sum_{j\neq i}\min\left[w_{ji},w_{ij}\right]$
Page-Rank	Social ranking	$\mathrm{Ran}k_{i}=\frac{1-d}{N}+d\sum_{j=1}^{n}\frac{w_{ij}\mathrm{Ran}k_{j}}{\sum_{k=1}^{n}w_{kj}}$

Average received cooperation, $r_{i}$, measures the average cooperation received by ego (i). Average sent cooperation, $s_{i}$, measures the average sent cooperation. Reciprocated weight, $R_{i}$, measures the average level of reciprocity for each ego (i). $\mathrm{Ran}k_{i}$, calculated using Page-Rank (Page et al., 1999), measures the relative social ranking of students based on the network of cooperation. See Supplementary Figure SM 3.1 for a correlation plot of all variables.

Evidence on the external validity of game-theoretic experiments (Karlan, 2005; Fehr and Leibbrandt, 2011; Gelcich et al., 2013; Hopfensitz and Miquel-Florensa, 2017; Algan et al., 2013; Rivera-Hechem et al., 2021) shows that real-life cooperation correlates with cooperation under laboratory conditions. Thus, we expect to capture students' cooperative dispositions towards their classmates as the behavior implemented in the experiment at the individual level. A distinctive feature of our approach compared to traditional game-theoretic experiments is that interactions are non-anonymous. We therefore anticipate capturing not just an individual's inherent propensity for cooperation but also their propensity for cooperation within their setting, shared narrative schemata of a specific relationship, and their shared history (Wang et al., 2017).

### 2.4 Difference-in-difference identification strategy

We use a difference-in-difference-like identification strategy to analyze the impact of reciprocity in academic performance that relies on the condition of the parallel trend for a causal interpretation of results. This condition indicates that to evaluate the effect of a given treatment (in this case, different levels of reciprocated cooperation), we should expect the evolution of different individuals to be the same in the absence of treatment.

It is noteworthy to consider some socio-economic configurations related to the educational context in Chile to evaluate the plausibility of unobserved time-invariant confounding variables in our model specification. First, most of the students have been in the same class group, i.e., with the same peers, for at least three years; spending around 8 h per day together. Second, the academic year (march to December) overlaps the fiscal year (January to December). Hence, we argue that students share a common past and a common history and that classroom and school-level changes are infrequent plausibly. At the individual level, the probability of a guardian's job changes, or a student's housing moving is very low within the fiscal year, mainly because these changes usually occur during holidays, between December and March, motivated by economic reasons.

Finally, we control for class attendance because some variables related to student engagement into school are not constrained by systemic configurations such as non-anticipated illnesses and family issues. Household income is also not constrained by systemic configurations; therefore, we control for the guardian's level of education as a proxy for household income. Thus, we also capture income variations.

## 3 Results

**Figure 2:**
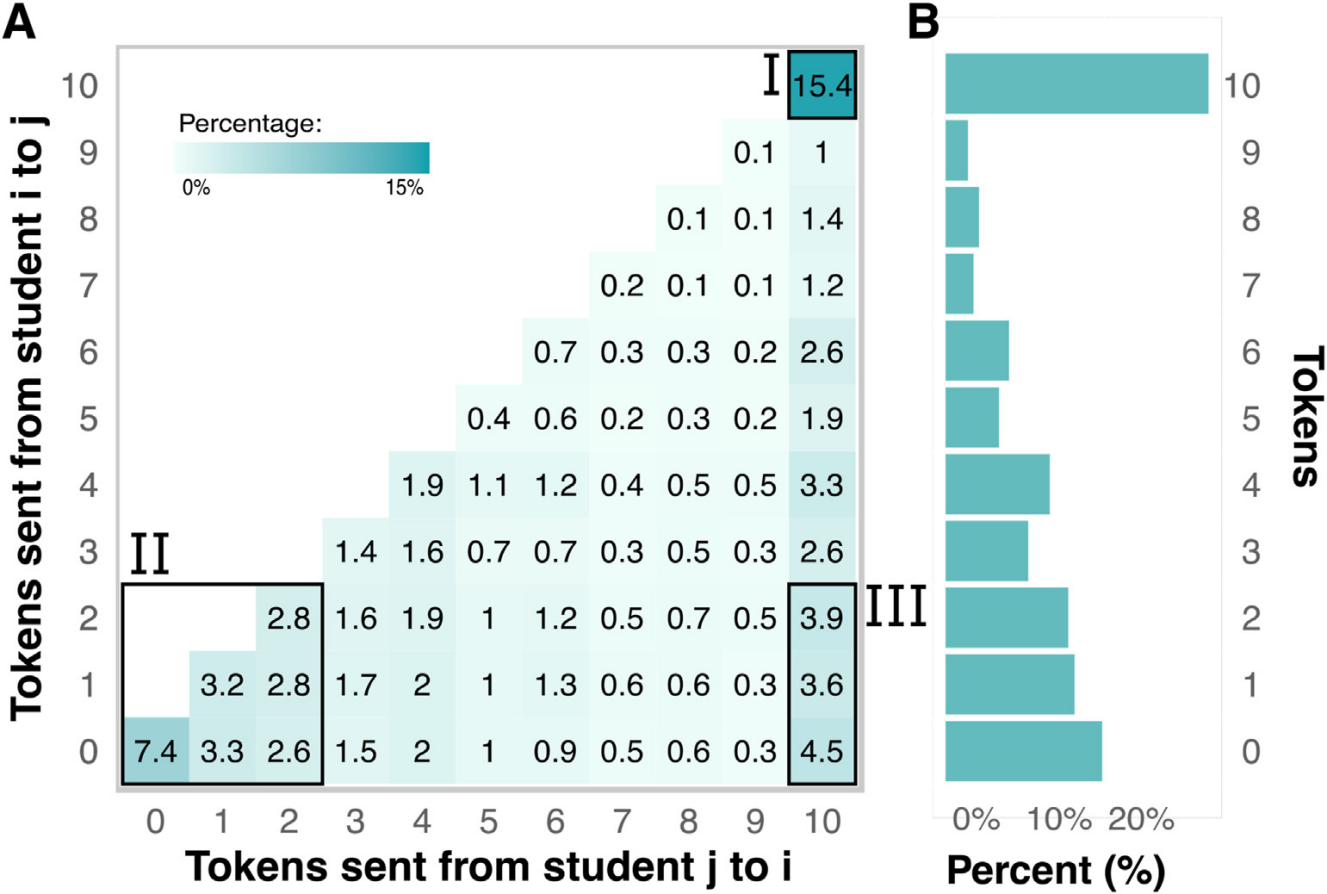
Thorough peer interactions' token sending and receiving patterns. (A) Bivariate sent token distribution. The figure shows three emerging clusters that illustrate the type of interaction between pairs of students: (I) Social optimum (top-right), where both students sent most of their tokens to each other. (II) Non-cooperative interactions (bottom-left), where both students did not send any or only a few tokens. (III) Asymmetric exchange (bottom-right), where one student sent most of their tokens, and the other sent only a few tokens. (B) Univariate distribution for sent tokens, showing the marginal distribution of Figure 2A. Although students could send any number of tokens between zero and ten, both panels show that students engaged in either fully cooperative (sending ten tokens, cluster I) or non-cooperative (sending two or fewer tokens, cluster II) strategies. The combination of both strategies leads to the emerging cluster III.

Figure 2 shows the emerging patterns of token sendings among students, where the total number of sending combinations among classmates is 18,334. Panel A shows the bivariate distribution of sent and received tokens. 15.4% of the interactions are fully cooperative (Figure 2A I), while 22.1% of interactions are highly defective and involve both students sending two or fewer tokens to each other (Figure 2B II). Asymmetric interactions are also visible in the behavioral game. A student sending 10 tokens and receiving two or less tokens in response occurs in around 12% of interactions (Figure 2B III). We also note that the sent tokens range spans from 0 to 10 in addition to pure cooperative strategies (always cooperate or always defect) (Supplementary igure 3.2), which suggest that common history and past is considered in the decision-making process (Wang et al., 2017).

Figure 2B depicts a histogram summarizing the amount of sent tokens. The figure shows that although students could send any number of tokens between zero and ten, they mainly engaged in either fully cooperative (sending ten tokens) or non-cooperative strategies (sending two or fewer tokens).

Now, we ask, do students who participate in a high number of mutually cooperative relationships increase their GPAs more than other students? We investigate if reciprocity, defined in each exchange as the minimum amount between sent and receiving tokens (see Table 1), plays a role in academic performance. Here, the ideal situation to correctly identify reciprocity's causal effect on academic performance would be to exploit an exogenous variation in reciprocity. Still, it is impossible to create such a variation in this type of experimental design due to the intricacies between reciprocity and peer interactions. Instead, we rely on statistical tools (Angrist and Pischke, 2008) to estimate the individual future GPA as a function of the individual-level average reciprocated cooperation, controlling for different confounders. Omitted variables simultaneously determining reciprocated cooperation and GPA improvement, or reverse causality could provide biased point estimations. Remarkable possible omitted variables affecting both academic performance and reciprocity are intelligence, illness, and socio-economic background (see Methods section for more detail); therefore, we provide proxy variables for them–prior academic performance, attendance, and guardian's education, respectively. However, we owledge that other unconsidered omitted variables could play a role in the identification process. Yet, our statistical tools help us unveil a cleaner effect of reciprocity on future GPA.

We rely on a two-fold identification strategy: first, refining the statistical model with relevant controls and a fixed effect controlling for average classroom characteristics. Second, we implement a complementary treatment intensity difference-in-differences estimation (Acemoglu et al., 2004) to address some concerns related to time-invariant unobserved confounders (See Methods Section).

We define the base statistical model (Eq. (1)) by estimating the individual future GPA as a function of the individual average reciprocated cooperation ($R_{i}$, see methods section and Table 1) as:

(1) $$GPA_{i1}=\beta_{1}R_{i}+\beta_{2}s_{i}+\beta_{3}\mathrm{Ran}k_{i}+\beta_{4}GPA_{i0}+\beta_{5}G_{i}+\beta_{6}A_{i}+\beta_{7}TESC_{i}+\theta_{c}+e_{i}\text{,}$$

where, $GPA_{i1}$ represents the GPA of the student i and $e_{i}$ is the error term. The effect of reciprocated cooperation could differ between students with different levels of sent tokens and social status within their classrooms. Also, both variables are arguably correlated with GPA (See SM3.1). To control for these sources of variation, we include the average number of sent tokens ($s_{i}$) and the PageRank ($\mathrm{Ran}k_{i}$)–a network measure to proxy individual social ranking computed using the full cooperation network (See Method sections and Table 1). We also included traditional confounding variables, such as gender ($G_{i}$), percentage of class attendance ($A_{i}$), level of education of the guardian ($TESC_{i}=1$ if the guardian completed secondary school, 0 otherwise), and fixed effects per classroom ($\theta_{c}$).

Table 2 displays the estimation for the model defined by Eq. (1), with a few variations in control variables. Column 1 shows the effect of reciprocated cooperation ($R_{i}$, presented as a z-score in the whole sample for interpretability) within each classroom. Using fixed effects, we control for the unobserved discrepancies among class groups $\theta_{i}$. We note that 5.4% of the explained variance ($R^{2}$ within) is due to the reciprocated cooperation within classrooms. However, to properly study reciprocated cooperation's effect and avoid omitted variable biases, we need to account for the individual average cooperation. By definition, a higher average cooperation leads to a higher reciprocated cooperation (see Table 1). We also need to control for individual social status ($\mathrm{Ran}k_{i}$) measured as the PageRank network centrality (Page et al., 1999; Bruch and Newman, 2018) (see Table 1) because students with higher social status will be the targets of more cooperation leading to an increase in their reciprocated cooperation. Therefore, column 2 shows our model controlling for sent cooperation and social rank, and we observe that the three variables are significant and explain 18.3% of the variation within the classroom. Finally, column 3 shows our model controlling for the traditional confounding variables. We note that the previous semester's GPA (Grades before measuring $GPA_{i0}$) quantifies previous individual accomplishments. Prior individual GPA provides a proxy for individual talent and controls by several time-invariant confounding variables that correlate with GPA, such as household income and practicing sports, among others. We also included attendance percentage, the education level of the student tutor, and students' sex. Thus, the total explained variance is 72.8% (See Supplementary SM 3.3 for a predicted v/s observed values plot for model 3 using both future GPAs as dependent variables).

**Table 2:** OLS regressions for students’ GPA one semester after measuring cooperation. Model 1 shows that reciprocity has a positive and significant effect on GPA and accounts for a 5.4% of the variance within students. Models 2 and 3 show that reciprocity between students significantly and positively affects GPA even after controlling for different control variables. Note that these models show results for 769 students because we do not have the data for Tutor complete secondary school for 86 students.

	Dependent variable: GPA (after measuring)		
	(1)	(2)	(3)
Reciprocated cooperation (z-score)	0.161∗∗∗	0.394∗∗∗	0.094∗∗∗
	(0.03)	(0.05)	(0.03)
Sent cooperation (z-score)		−0.285∗∗∗	−0.063∗∗
		(0.04)	(0.03)
Rank (z-score)		0.145∗∗∗	0.065∗∗∗
		(0.04)	(0.02)
Grades (before measuring)			0.654∗∗∗
			(0.02)
Attendance (%)			0.010∗∗∗
			(0.00)
Tutor comp. sec. school (yes)			0.019
			(0.02)
Sex (Male)			−0.055∗∗
			(0.03)
Fixed effects	Class-group	Class-group	Class-group
Observations	769	769	769
R^2^	0.268	0.368	0.746
Adjusted R^2^	0.222	0.327	0.728
R^2^ within	0.054	0.183	0.672
F Statistics	41.079	54.010	209.438

*Note:* ∗p < 0.1; ∗∗p < 0.05; ∗∗∗p < 0.01.

We find a positive and significant effect of reciprocated cooperation on GPA. More specifically, we show that an increase in reciprocal cooperation of one standard deviation is linked to a future GPA rise of 0.094 units. We point out that the scale for grades in Chile ranges from 1 to 7, and that the average grades for the first and second semesters under consideration are 5.87±0.58 and 5.79±0.57, respectively. Therefore, the average variation between both semesters is $\Delta_{GPA}=-0.080$. Thus, the reciprocated cooperation effect size (0.094) and the average decrease of GPA between the two periods (−0.080) are comparable. Indeed, the effect size is 117.5% of the average GPA variation between semesters.

Yet, some meaningful unobserved confounding variables could be affecting our results. For instance, modifications to the composition of the classroom, such as the addition of new instructors and pupils or a rise or fall in school funding. Also, changes at the individual level, such as a new job for the student's guardian or a house move, would impact household income and social capital outside of the classroom, respectively. However, given the Chilean educational context, we can reasonably assume that most of these unobserved variables are time-invariant within our study period spanning July to December 2017 (for more details, see Methods Section).

Thus, to overcome all potential issues related to time-invariant unobserved confounders and provide evidence on the magnitude of the relationship between reciprocated cooperation and GPA improvement, we use a treatment intensity difference-in-difference framework (Acemoglu et al., 2004). Individual average reciprocal cooperation $R_{i}$, a continuous quantity that induce fluctuation at the individual level, serves as our treatment intensity variable in our approach. Our model is specified as follows:

(2) $$GPA_{it}=\beta_{1}+\beta_{2}T+\beta_{3}R_{i}+\beta_{4}A_{it}+\delta T\times R_{i}+\varepsilon_{i}+e_{it}\text{,}$$

where $\varepsilon_{i}$ represents individual-level fixed effects (individual-level fixed effects are seen to absorb classroom-level fixed effects.) and $e_{it}$ is the error term. $GPA_{it}$ is the GPA of student i in period t, T represents the semester and it takes values 0 (before measuring, t=0) and 1 (after measuring, t=1), $R_{i}$ is the treatment intensity (reciprocated cooperation), $A_{it}$ is the class attendance for both time periods, and finally, the diff-in-diff estimator is represented by δ. Table 3 shows four variations of the specification in Eq. (2).

**Table 3:** Difference-in-difference estimation of GPA. Models 1 and 2 differ in the control variable “class attendance” and consistently show reciprocity's positive and significant effect between two periods of GPA measurement. We created a dummy variable for reciprocated collaboration in Models 3 and 4 that takes the value 1 if person i's degree of reciprocated cooperation is in the top 20% and the value 0 otherwise. Difference-in-difference models confirm previous results (Table 2) even after controlling for time-invariant unobserved confounders. Models also confirm the presence of heterogeneity in the effect of reciprocity, where the effect is more prominent in students among the top percentiles of reciprocity in each classroom.

	Dependent variable: GPA			
	(Diff-in-Diff)			
	(1)	(2)	(3)	(4)
Reciprocity ∗ Time	0.039∗∗∗	0.038∗∗∗		
	(0.01)	(0.01)		
Time	−0.081∗∗∗	−0.094∗∗∗	−0.102∗∗∗	−0.114∗∗∗
	(0.01)	(0.01)	(0.01)	(0.01)
Attendance (%)		0.008∗∗∗		0.008∗∗∗
		(0.00)		(0.00)
Top 20% of reciprocity ∗ Time			0.106∗∗∗	0.100∗∗∗
			(0.03)	(0.03)
Fixed effects	Individual	Individual	Individual	Individual
Observations	1710	1710	1710	1710
Students	855	855	855	855
R-squared	0.894	0.897	0.894	0.897
Adjusted R-squared	0.787	0.793	0.787	0.793
R-squared within	0.054	0.082	0.056	0.083
F Statistics	24.535	25.217	25.504	25.604

*Note:* ∗ p< 0.1; ∗∗∗p < 0.05; ∗∗∗p < 0.01.

Thus, we conclude that our diff-in-diff estimator is reliable and consistent with the models in Table 2. Particularly interesting is model 2 in Table 3. The diff-in-diff estimator is positive (0.038) and significant (p-value <0.01), above and beyond the effects of the unobserved time-invariant individual characteristics (individual fixed effects). Our diff-in-diff estimation's size effect (Model 2 Table 3) is about 40% larger than the associative effect seen in Table 2 model 3.

In model 4 Table 3, we set a dummy variable for the reciprocated cooperation as 1 for all individuals in the top 20% of reciprocated cooperation and 0 otherwise. We observe a higher size effect of reciprocated reciprocity (0.100), suggesting that the effect is heterogeneous in the reciprocated reciprocity range. Indeed, as a robustness check, we estimate the effect of reciprocity on GPA by setting nine dummies for different thresholds of reciprocity, ranging from the bottom-10% to the top-10% of reciprocated cooperation. Although the average effect of reciprocity is 0.038 (Table 3 Model 2), we found that the effect of reciprocity increases from the median to the top 10% reciprocity, confirming that the effect is more substantial for students over the top-20% of reciprocated cooperation.

## 4 Discussion

Individuals’ position in their social networks is associated with educational outcomes at all ages (Baldwin et al., 1997; Caprara et al., 2000; Bruun and Brewe, 2013; Gašević et al., 2013; Blansky et al., 2013; Ivaniushina and Alexandrov, 2018; Stadtfeld et al., 2019; Kassarnig et al., 2018; Berthelon et al., 2019; Pulgar et al., 2020; Candia et al., 2022a, Candia et al., 2022b; Pulgar et al., 2022a, Pulgar et al., 2022b). The social network position and academic achievement of a student are also said to be positively correlated in the social learning literature. There are three possible and non-exclusive explanations in this context: (i) central students get better GPAs through positive learning externalities from their social connections; (ii) higher GPA leads to a higher status, resulting in more central students in their social networks; (iii) more talented students are more strategic in their interactions and learn faster, leading to increases in GPA that expand the gap with less talented students. Here, we provide evidence for the first explanation by studying cooperative patterns in elementary school students using a video game based on game theory and a difference-in-difference identification strategy.

To overcome the potential biases in survey measurement instruments, we implemented a lab-in-the-field approach to map students' social relationships in their classrooms. Students played a non-anonymous social dilemma on interconnected tablet computers through a friendly user interface where they had to choose how many tokens to share with their peers (Figure 1A). Thus, we mapped the entire student's cooperative network in their classroom (Figure 1B). We found that students mainly engage in three types of cooperative relationships: fully cooperative (Figure 2, I), non-cooperative (Figure 2, II), and relationships in which the cooperation is asymmetric (Figure 2, II).

Then, we define reciprocated cooperation (Table 1) as the minimum between sent and received cooperation and found it improves elementary school students' GPAs. We provided evidence on the positive and significant effect of reciprocated cooperation and GPA using both linear models (Table 2) and a difference-in-difference identification strategy (Table 3) exploiting an endogenous treatment intensity variable (Acemoglu et al. 2004). In the former, we show that the effect of received, sent, and reciprocated tokens survives even after controlling by confounding variables, such as previous GPA, sex, class attendance, and the educational level of the student's guardian. We find evidence supporting reciprocated cooperation as a predictor of future GPA. In the latter, the main premise is that, in the absence of our treatment intensity variable, the reciprocal collaboration, both the control and treatment groups exhibit comparable patterns. In other words, when students don't reciprocally cooperate, changes in GPA tend to follow a similar pattern. Of course, there are certain restrictions here. Any change within the time interval studied in a confounding variable that affects both GPA and reciprocated cooperation could hinder our results. For instance, a change in family income may impact reciprocity through the popularity of a student. Also, it may affect GPA through getting a private professor or changes in access to the internet (Jackson et al., 2006). Also, practicing sports may impact reciprocated cooperation through popularity, and it may affect GPA (Singh et al., 2012; Rees and Sabia, 2010) at getting healthy. Thus, any change between the first and second semester of the academic year in which the experiment was implemented in the level of a guardian's student income or the student started to practice a sport could have impacted reciprocated cooperation and GPA. These changes would make the parallel trend assumption for our difference in difference specification unfulfilled because we cannot control or measure any of these changes. It is never feasible to guarantee that the parallel trends assumption is satisfied in its entirety. However, assuming the parallel trend holds in our context (see Method Section), we present strong and consistent evidence of a directional impact from reciprocal cooperation to GPA improvement. On the other hand, by controlling for unobserved time-invariant individual characteristics, we already account for all features that arguably do not change from one semester to the next such as talent. Therefore, a different statistical setting is needed to explore the role of talent stated in the third explanation mentioned before.

We found that the three social network measures–Social Rank, Sent cooperation, and Reciprocated cooperation (see Table 1)–account for 18.3% of the variance within classrooms, where 5,4% is given by Reciprocated reciprocity (Table 2). The explained variance remains the same even after controlling for all time-invariant confounders (Table 3). As a robustness check, we explored the heterogeneity of the effect of reciprocity on GPA, and we found that the effect of reciprocity increases from 0.039 to 0.100 for students belonging to the top-20% of reciprocated cooperation.

We consider this work to contribute to our understanding of the link between social networks and learning outcomes and how novel methodologies such as experimental game theory can help us in this endeavor.

From a methodological standpoint, our findings provide new opportunities for the application of game-theoretical and network-based methods to harness relational information in primary education (Burt, 2000; Lakon et al., 2008) while avoiding conventional survey instrument biases. We propose a measure of the individual's social capital, which is directly represented in the connections between students and indirectly by the configuration of the network as a whole. This helps to improve the external validity of game-theoretic experiments in the setting of schools (Lakon et al., 2008). Using this approach, we can map a representative social network for a group of young people with a common history (Wang et al., 2017). The generalizability of our results should be qualified. Previous experimental evidence in non-WEIRD societies shows that similar patterns of behavior emerge in different societies and cultures, e.g., in most societies, people reject unfair offers in the Ultimatum Game. However, the modal threshold for rejection varies across different societies (Henrich et al., 2005; Ensminger and Henrich, 2014). Similarly, one could expect that the reciprocal dispositions observed in our Chilean primary school classrooms sample will also be observed in different contexts, but their prevalence could vary. Further research from a comparative perspective would be required to study this variation and its subsequent effect on academic performance.

Finally, from a policy perspective, the natural question to ask is what kind of intervention might improve students’ academic performance by optimizing the potential benefits of cooperation? Our results, together with the lack of GPA homophily (see Supplementary Notes SM 1.1), suggest that encouraging social relationships through, for instance, interventions of the spatial arrangement of the class (Salend, 1999), fostering community-school partnership (Haines et al., 2015; Ratcliff and Hunt, 2009), an instructional design that promotes the formation of social ties in the classroom (McFarland et al., 2014; Rohrbeck et al., 2003; Slavin, 2011, 2011; Newman, 2002; Slavin et al., 2003; Pulgar et al., 2019; Pulgar et al. 2020; Pulgar et al., 2022a, Pulgar et al., 2022b), or any other intervention that aims to support cooperation in classrooms (Nowak, 2006; Axelrod and Hamilton, 1981; Trivers, 1971; Nowak and Sigmund, 2005; Sigmund et al., 2001) are potentially fruitful alternatives to explore. Thus, we open the possibility for intervention by promoting relationships within the classroom that might significantly affect the academic achievements of primary students. We must note that our study was conducted before the COVID-19 pandemic. Therefore, we cannot study the role of reciprocity on academic performance in a remote or online class setting. However, our results could inspire and inform new research whose aim is to maximize work-group compositions in remote or online learning environments.

## Declarations

### Author contributions statement

Cristian Candia conceived and designed the experiments; performed the experiments; analyzed and interpreted the data; contributed materials, analysis tools, and data; wrote the paper.

Melanie Oyarzún analyzed and interpreted the data; wrote the paper.

Victor Landaeta performed the experiments; analyzed and interpreted the data.

Tamara Yaikin performed the experiments.

Cecilia Monge conceived and designed the experiments.

César Hidalgo analyzed and interpreted the data.

Carlos Rodriguez-Sickert conceived and designed the experiments; interpreted the data; wrote the paper.

### Funding statement

Cristian Candia was supported by ANID FONDECYT Iniciación112-00986 and Carlos Rodriguez-Sickert was supported by ANID FONDEF IT15I10079.

### Data availability statement

Data will be made available on request.

### Declaration of interest's statement

The authors declare no conflict of interest.

### Additional information

No additional information is available for this paper.

## Appendix A Supplementary data

SM_reciprocity_improves_GPA_children.pdf SM_reciprocity_improves_GPA_children.pdf
