# Supplementary material for: Reciprocity heightens academic performance in elementary school students
Source: Heliyon. 2022 Dec 5;8(12):e11916. doi: 10.1016/j.heliyon.2022.e11916 (PMC9763753; doi:10.1016/j.heliyon.2022.e11916)
Supplement: SM_reciprocity_improves_GPA_children.pdf [file mmc1.pdf]

Supplementary Material
Reciprocity heightens academic performance in elementary school students

Cristian Candia[1,2,*], Melanie Oyarzún[3], Victor Landaeta[3], T. Yaikin[3], C. Monge[4], César Hidalgo[5,6,7], and C. Rodriguez-Sickert[3]

[1]Data Science Institute, Facultad de Ingeniería, Universidad del Desarrollo, Las Condes, 7610658, Chile.

[2]Northwestern Institute on Complex Systems (NICO), Northwestern University, Evanston, IL 60208, USA

[3]Centro de Investigación en Complejidad Social (CICS), Facultad de Gobierno, Universidad del Desarrollo, Chile.

[4]Feedback comunicaciones, Vitacura, Chile.

[5]ANITI Chair, University of Toulouse, Toulouse, France

[6]Alliance Manchester Business School, University of Manchester, Manchester, UK

[7]School of Engineering and Applied Sciences, Harvard University

[*]Corresponding Authors: cristiancandia@udd.cl

November 9, 2022

Supplementary Notes

SM 1.1: Homophily Index for GPA

Our proposed approach for mapping the social capital network in elementary classrooms unveils the non-existence of homophily between students regarding GPA. We calculate the homophily index [?] as follow:

$$H_r = corr\left(G_i^r, \frac{1}{N^r}\sum_j^{N^r} G_j^r\right), \tag{S1}$$

where G_{ir} represents the GPA of the ego student "i" and G_{jr} represents the GPA of the alter student "j". N indicates the number of students with whom the ego shares the same reciprocity level, r. The superindex r represents the level of reciprocity in the interaction. Those levels are: i) Low reciprocity (0.0) includes the interactions where egos and alters send fewer tokens than their average amount sent to the rest of their class. ii) Positive asymmetry (0.1) includes the interactions where alters send more tokens than their average sent tokens and egos send fewer tokens than their average sent amount, regarding their class. iii) Negative asymmetry (1.0) includes the interactions where egos send more tokens than their average sent tokens and alters send fewer tokens than their average sent amount,

regarding their class. iv) High reciprocity (1.1) includes the interactions where egos and alters send more tokens than their average amount sent to the rest of their class.

Fig. S1 shows that in the "Cooperation Network" most of the classes have Homophily Indexes non-significantly different from zero, with both GPA before and after measuring. This finding is interesting because it has been shown that GPA modulates social relationship in older students–High School, undergrads, and grad students–[**?**, **?**, **?**], however, here we provide evidence on this behavioral pattern does not manifest in elementary school students. Thus, interventions to promote and boost cooperative social relationships could be possible to improve academic performance, by exposing students with low GPA to the idea flow and information of the students with high GPA [**?**].

These results implies that cooperative interactions in children do not seem to be driven by academic homophily.

Table S1: Homophily Index of Cooperation Networks on GPA before and after measuring, by Class

Class ID	Homophily Index GPA (Before)	Homophily Index GPA (After)
10	-0.1947	0.0042
11	-0.1993	-0.3993
12	0.2509	0.5652 **
13	-0.2539	-0.3811
14	0.8272 ***	0.7763 ***
15	0.5044	0.0851
16	-0.2429	-0.2542
17	-0.2446	-0.0952
18	0.0499	-0.2112
19	0.0986	-0.1119
20	-0.0348	0.0633
21	0.0708	0.1366
23	0.0893	0.5436 ***
24	-0.1848	0.2128
25	-0.311	-0.456 **
26	-0.0834	-0.3109
27	-0.1472	-0.1304
28	0.1729	0.462 **
29	-0.5273 **	-0.2093
30	-0.3425	-0.4028
31	-0.2936	0.1011
32	-0.2142	-0.1654
33	0.3756 *	-0.1863
34	-0.8316 ***	-0.2617
35	0.5746 *	-0.2938
38	-0.1215	0.2298
39	-0.2444	-0.48 *
40	0.3102	0.6662 ***
41	0.0607	0.1991
42	-0.5799 *	-0.3401
43	0.045	-0.4457
44	-0.1497	-0.0175
45	-0.09	-0.5937
46	-0.5 **	-0.305
47	-0.6942 *	-0.6366
48	-0.8405 **	-0.6899
49	-0.3657	-0.1622
50	0.2362	0.2207
51	-0.0339	-0.1241
52	0.2206	0.4032 *
55	-0.0127	0.0591
56	-0.0086	0.4444 *
57	-0.454 **	-0.3179
58	-0.1344	0.0492
59	0.1459	-0.5666 **

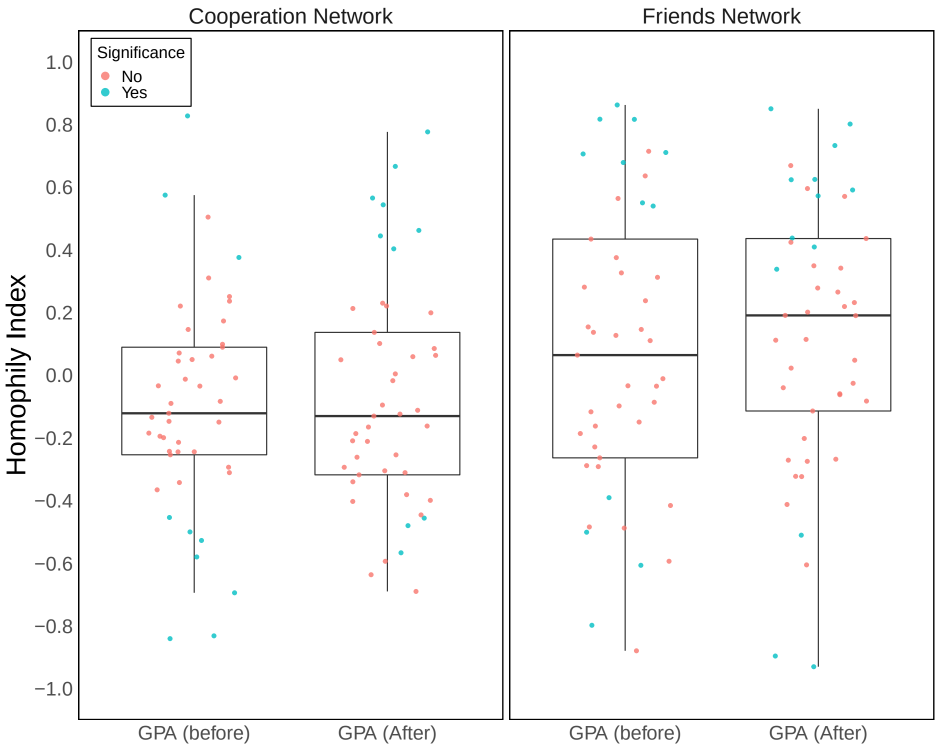

Figure S1: Homophily index according to GPA in elementary school students. The homophily index for high reciprocity is non-significantly different from zero, which means that elementary students do not build their cooperative disposition based on the GPA of their peers. The significance is given by color, and it represents a p-value under 0.1. Most of the rest of the homophily coefficients are low if we compare them with the literature for older students [**?**], where the initial level of homophily is greater than 0.2 and evolves until 0.4.

Supplementary Tables

SM 2.1: Classrooms descriptions

Table S2: Class-level description of experimental data.

Class ID	School ID	Age (Mean)	Age (SD)	Female (%)	Guardian education Secondary school completed (%)	GPA (Mean)	Class size	Attendance (Mean %)	Cooperation (Mean tokens)
1	1	10.03	0.57	57.6	48.5	6	33	89.3	3.97
2	2	9.09	0.51	41.7	45.8	6	24	91.9	5.59
3	2	10.17	0.51	53.3	56.7	5.9	30	90.2	4.76
4	2	10.87	0.66	43.5	56.5	5.9	23	89.2	4.39
5	3	9.15	0.99	30.8	30.8	5.7	13	89.2	6.09
6	3	9.05	0.77	56.2	50	5.6	16	93.3	5
7	3	10.39	0.9	39.1	34.8	5.8	23	91.6	4.86
8	3	10.91	0.7	33.3	50	5.7	18	93.7	4.3
9	3	11.46	1.3	52.6	52.6	5.2	19	88.9	7.14
10	4	9.17	0.64	37.5	50	6	8	94.6	7.23
11	4	10.01	0.95	42.9	28.6	6	7	97.5	4.88
12	4	11.06	0.77	42.9	38.1	6.1	21	88.9	5.1
13	5	10.05	0.72	55	30	6.6	20	90.3	5.04
14	5	10.18	0.78	58.8	35.3	6	17	91	4.21
15	5	11.22	0.63	50	30.8	5.8	26	93.7	3.54
16	6	9.41	1.05	66.7	55.6	5.9	9	92	6.25
17	6	10.3	0.95	33.3	50	5.5	12	88.3	5.92
18	6	11.66	2.56	35.7	64.3	5.8	14	89.6	4.06
19	7	9.17	0.74	73.3	26.7	6.1	15	95.2	4.38
20	7	10.3	0.84	47.6	28.6	6	21	95.3	5.9
21	7	11.24	0.73	33.3	44.4	5.8	18	94.4	4.63
22	8	9.2	0.64	46.2	43.6	5.7	39	89.8	5.08
23	8	10.11	0.64	63.6	42.4	5.9	33	92.4	4.06
24	8	11.12	0.55	48.4	45.2	5.4	31	91.2	4.34
25	9	8.98	0.63	100	44	6.2	25	88.8	6.78
26	9	9.87	0.76	100	35.7	6	14	83.7	5.32
27	9	10.73	0.65	100	50	6	14	89.4	5.31
28	10	9.07	0.92	36.4	22.7	5.7	22	86.8	3.79
29	10	9.38	1.12	61.5	30.8	5.7	13	91.3	7.22
30	10	10.12	0.59	45.5	22.7	5.8	22	85.2	3.48
31	10	10.35	0.95	50	22.7	5.6	22	84.6	4.05
32	10	11.44	1.08	53.6	25	5.7	28	88.3	3.79
33	11	8.8	0.52	0	50	5.7	14	90.7	6.81
34	11	10.3	0.43	0	75	6	12	94.3	3.92
35	11	11	0.67	0	50	6.2	10	94.2	3.09
36	12	9.47	0.83	58.3	25	5.8	12	90.8	5.59
37	12	10.2	0.88	20	30	5.6	10	87.9	4.39
38	12	11.65	1.11	35.3	47.1	5.5	17	89.3	3.2
39	13	9.92	0.5	100	69.2	6.1	26	94.2	3.62
40	13	9.97	0.49	100	38.9	5.8	18	91.8	4.67
41	13	9.8	0.61	100	70	6.2	20	94.8	5.29
42	14	8.91	0.61	100	72.2	6.3	18	96	5.33
43	14	8.97	0.69	100	50	6	18	91.6	5.6
44	14	10.04	0.56	100	63.2	6	19	91	5.11
45	14	11.7	1.64	100	73.3	6.2	15	96.2	4.09

Supplementary Figures

SM 3.1: Correlation Matrix

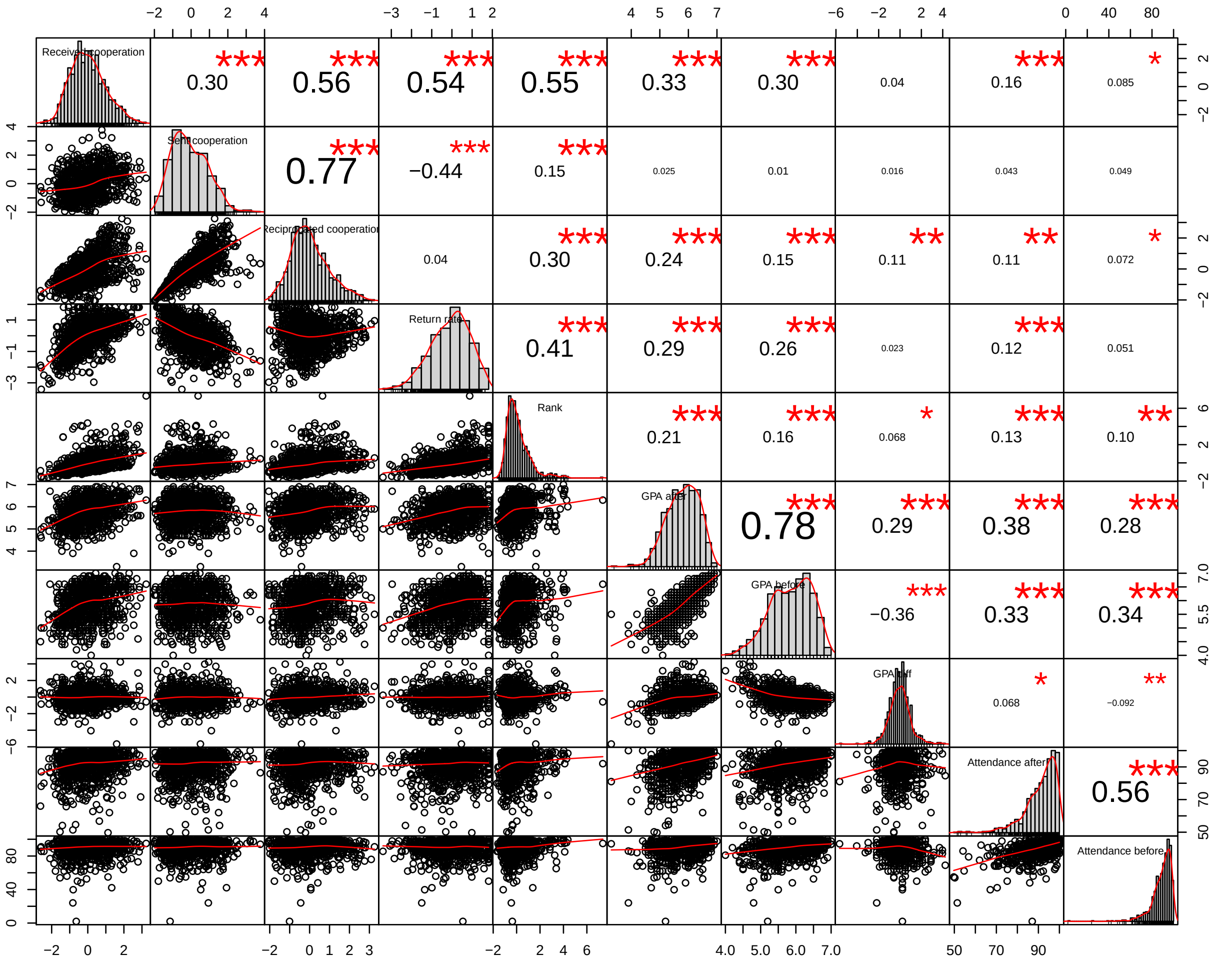

Figure S2: Correlations between all collected, measured, and built variables.

SM 3.2: Sent tokens by students

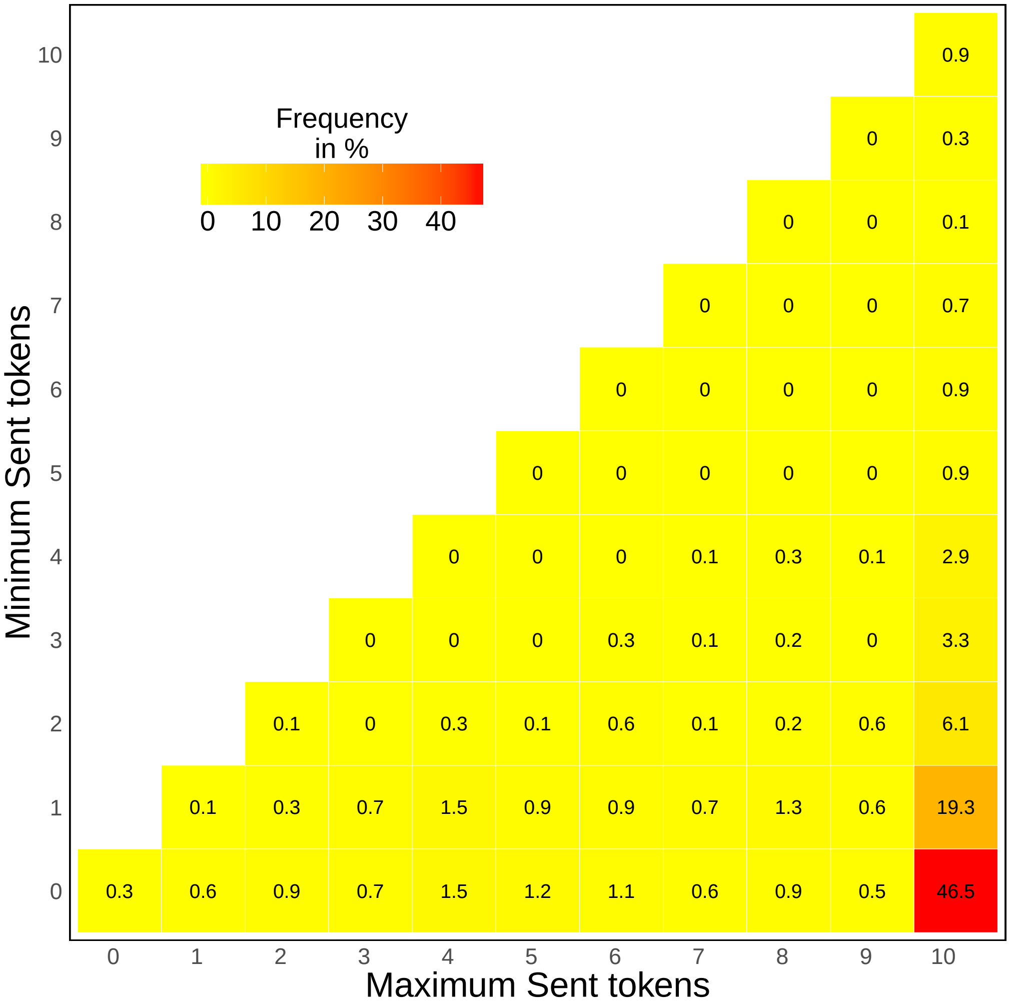

Figure S3: Heatmap for maximum and minimum sending. At the bottom-right corner, we observe a cluster (≈ 65% of students, red/orange blocks) indicating that students send tokens in the whole range of possibilities. History between colleagues matters.

SM 3.3: Explanatory Power of the Model

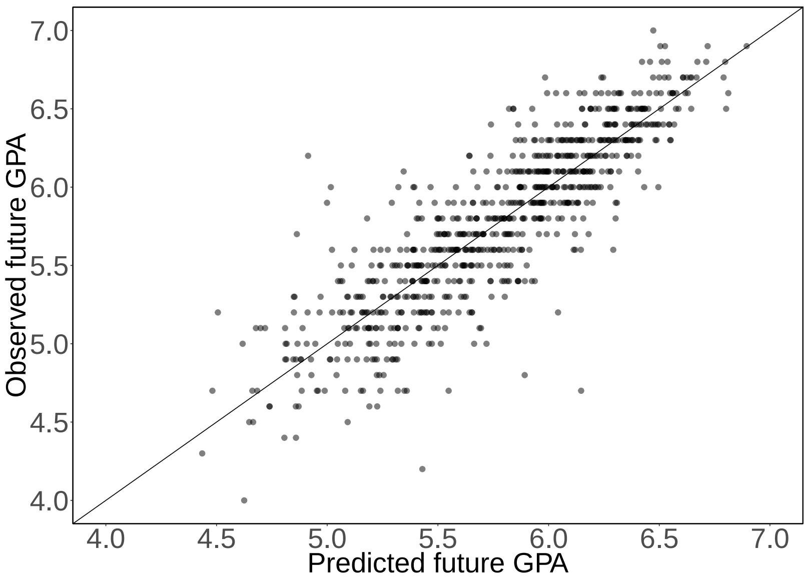

Figure S4: Observed v/s predicted GPA (table 2 model 3)

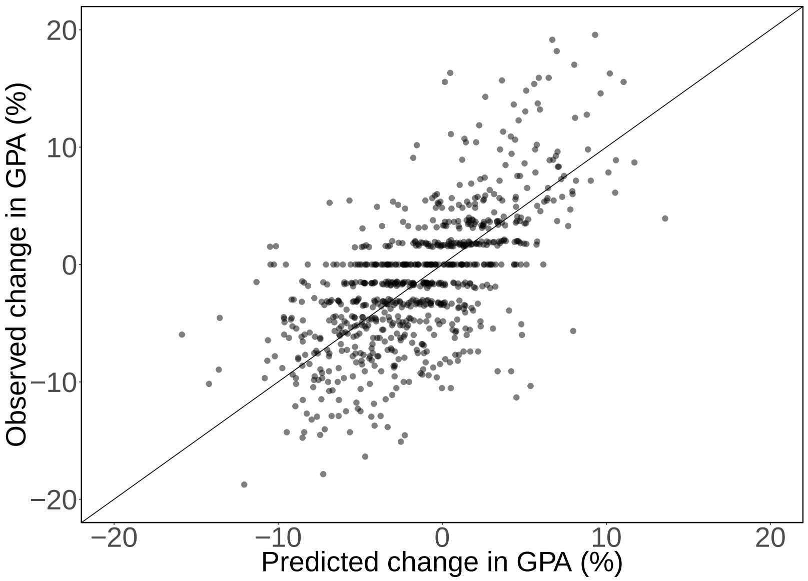

Figure S5: Observed v/s predicted change in GPA .

SM 3.4: Reciprocity and confounders

	Dependent variable:						
	Reciprocity (z-score)						
	(1)	(2)	(3)	(4)	(5)	(6)	(7)
Sent cooperation (z-score)	0.712***		0.706***			0.705***	0.705***
	(0.02)		(0.01)			(0.02)	(0.02)
Rank (z-score)		0.449***	0.425***			0.343***	0.343***
		(0.05)	(0.02)			(0.02)	(0.02)
Grades (before measuring)				0.255***	0.207***	0.158***	0.158***
				(0.05)	(0.05)	(0.03)	(0.03)
Attendance (%)					0.008**	0.003*	0.003*
					(0.00)	(0.00)	(0.00)
Tutor comp. sec. school (yes)					0.018	0.037	0.037
					(0.06)	(0.03)	(0.03)
Sex (Male)					-0.166***	-0.119***	-0.119***
					(0.06)	(0.03)	(0.03)
Constant	-0.007	-0.003	-0.007	-1.499***	-1.883***	-1.172***	-1.172***
	(0.02)	(0.02)	(0.01)	(0.29)	(0.38)	(0.19)	(0.19)
Fixed effects	Class-group	Class-group	Class-group	Class-group	Class-group	Class-group	Class-group
Observations	859	859	859	859	771	771	771
R-squared	0.815	0.507	0.868	0.465	0.489	0.883	0.883
Adjusted R-squared	0.804	0.480	0.861	0.435	0.455	0.875	0.875
R-squared within	0.665	0.108	0.761	0.032	0.053	0.784	0.784
F Statistics	1610.971	98.361	1294.442	26.729	10.085	435.206	435.206

Note: *p<0.1; **p<0.05; ***p<0.01

Table S3: OLS Estimation of Reciprocity (class-group fixed effects)

SM 3.5: Robustness of difference in difference estimation

	Dependent variable: Reciprocity (Diff-in-Diff)										
	(1)	(2)	(3)	(4)	(5)	(6)	(7)	(8)	(9)	(10)	(11)
Reciprocity * Time	0.039***	0.038***									
	(0.01)	(0.01)									
Time	-0.081***	-0.094***	-0.086***	-0.087***	-0.080***	-0.075***	-0.069***	-0.125***	-0.113***	-0.114***	-0.105***
	(0.01)	(0.01)	(0.01)	(0.01)	(0.02)	(0.02)	(0.02)	(0.02)	(0.02)	(0.01)	(0.01)
Attendance (%)		0.008***	0.008***	0.008***	0.008***	0.008***	0.008***	0.008***	0.008***	0.008***	0.008***
		(0.00)	(0.00)	(0.00)	(0.00)	(0.00)	(0.00)	(0.00)	(0.00)	(0.00)	(0.00)
Bottom 10% Reciprocity * Time			-0.089**								
			(0.04)								
Bottom 20% Reciprocity * Time				-0.037							
				(0.03)							
Bottom 30% Reciprocity * Time					-0.050*						
					(0.03)						
Bottom 40% Reciprocity * Time						-0.048*					
						(0.03)					
Bottom 50% Reciprocity * Time							-0.051**				
							(0.03)				
Top 40% Reciprocity * Time								0.075***			
								(0.03)			
Top 30% Reciprocity * Time									0.060**		
									(0.03)		
Top 20% Reciprocity * Time										0.100***	
										(0.03)	
Top 10% Reciprocity * Time											0.094**
											(0.04)
Fixed effects	Individual	Individual	Individual	Individual	Individual	Individual	Individual	Individual	Individual	Individual	Individual
Observations	1710	1710	1710	1710	1710	1710	1710	1710	1710	1710	1710
R-squared	0.894	0.897	0.896	0.896	0.896	0.896	0.896	0.897	0.896	0.897	0.896
Adjusted R-squared	0.787	0.793	0.791	0.791	0.791	0.791	0.791	0.792	0.792	0.793	0.792
R-squared within	0.054	0.082	0.077	0.073	0.075	0.076	0.076	0.081	0.077	0.083	0.078
F Statistics	24.535	25.217	23.584	22.506	23.164	23.254	23.434	24.993	23.727	25.604	23.900

Note: *p<0.1; **p<0.05; ***p<0.01

Table S4: Diff-Diff Estimation of Reciprocity (individual fixed effects)
